# Management of central nervous system relapse in a young patient affected by primary mediastinal large B‐cell lymphoma: A case report

**DOI:** 10.1002/ccr3.2706

**Published:** 2020-04-08

**Authors:** Miriam Marangon, Beatrice Casadei, Alessandro Broccoli, Lisa Argnani, Michele Cavo, Pier Luigi Zinzani

**Affiliations:** ^1^ Institute of Hematology “L. e A. Seràgnoli” University of Bologna Bologna Italy

**Keywords:** autologous stem cell transplant, central nervous system involvement, MATRix regimen, primary mediastinal large B‐cell lymphoma

## Abstract

In primary mediastinal large B‐cell lymphoma, central nervous system (CNS) relapse is an uncommon event with a dismal prognosis. We report about the successful management of CNS relapse with chemoimmunotherapy according to MATRix (methotrexate, cytarabine, thiotepa, and rituximab) protocol followed by autologous stem cell transplant.

## Graphical abstract

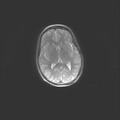

## 1 INTRODUCTION

Primary mediastinal large B‐cell lymphoma (PMBCL) is an uncommon tumor which constitutes about 2%‐3% of all non‐Hodgkin lymphomas and 6%‐10% of diffuse large B‐cell lymphomas (DLBCL). In this disease, central nervous system (CNS) relapse is a quite uncommon event, which constitutes nevertheless a therapeutic challenge due to its dismal prognosis. Since in literature there are currently no data available concerning management of CNS relapse in PMBCL, we refer to studies conducted in DLBCL. Here, we report about the successful management of CNS relapse with chemoimmunotherapy according to MATRix (methotrexate, cytarabine, thiotepa, and rituximab) protocol followed by autologous stem cell transplant in a young woman affected by PMBCL. Further follow‐up is needed to determine long‐term outcome.

Primary mediastinal large B‐cell lymphoma (PMBCL) is a mature B‐cell neoplasm which arises from thymic B cells.^1^ This uncommon disease affects more often women in their third or fourth decades of life. It usually presents with a rapidly progressive mediastinal mass which can be associated with symptoms related to superior vena cava syndrome. About 70% of patients have a bulky mass (>10 cm) at diagnosis. Distant spread to extra‐mediastinal lymph nodes or extranodal sites (ie, to the kidneys, adrenal glands, liver and ovaries) is uncommon at the time of presentation, but it can occur more frequently at the time of relapse.^2^, ^3^ Central nervous system (CNS) involvement rarely occurs at the time of onset, and it can be a quite infrequent site of disease recurrence; the main pattern of CNS disease is constituted by parenchymal lesions.^4^, ^5^ CNS recurrence is typically characterized by a dismal prognosis, and its treatment represents a difficult challenge for clinicians.

Here, we report about the successful management of CNS relapse with chemoimmunotherapy according to MATRix (methotrexate, cytarabine, thiotepa, and rituximab) protocol followed by autologous stem cell transplant (ASCT) in a young patient affected by PMBCL.^6^, ^7^

A 25‐year‐old young woman was transferred to our Institute in October 2016 from emergency department, where she was admitted due to the appearance of hacking cough, facial edema and conjunctival swelling. She had undergone a total body computed tomography (CT) scan, which showed a right mediastinal bulky mass (transverse diameters of 11 and 7.5 cm, respectively, and longitudinal diameter of 10 cm) compressing right cardiac structures, inferior vena cava, right pulmonary artery, right bronchus, surrounding ascending aorta and infiltrating pleura. Furthermore, supra and subdiaphragmatic adenopathies, multiple pulmonary nodules, a right pleural effusion, pancreatic, renal and right adrenal involvement were observed. Subsequently, a CT‐guided mediastinal biopsy was performed, leading to a diagnosis of PMBCL. Immunophenotype on diagnostic biopsy was as follows: CD20+, PAX5+, CD30±, CD10‐, BCL6+, BCL2±, IRF4+. ^18^F‐fluorodeoxyglucose positron emission tomography (^18^FDG‐PET) confirmed the presence of uptake at the mediastinal bulky mass, further mediastinal adenopathies, lungs, pancreas, kidneys, and right adrenal gland. Bone marrow biopsy was negative. According to Ann Arbor staging system, the disease was stage IV A, due to the absence of lymphoma‐related “B” symptoms. Physical examination revealed only the presence of neck and shoulders' edema; peripheral blood count revealed mild anemia and a white blood cell count of 13.000/mmc, with a normal differential count; platelet count was normal. Lactate dehydrogenase (LDH) was elevated. Age‐adjusted IPI was 2 (high‐intermediate risk).

From October 2016 to January 2017, the patient was treated with 12 cycles of R‐MACOP‐B chemoimmunotherapy, which is administered weekly (cyclophosphamide and doxorubicin on cycles 1, 3, 5, 7, 9, 11; methotrexate and vincristine on cycles 2, 6, 10; bleomycin and vincristine on cycles 4, 8, 12) with prednisone given continuously and anti‐CD20 rituximab boost during four of the 12 cycles. During the treatment, the patient experienced grade IV neutropenia responsive to granulocyte‐colony stimulating factor, methotrexate‐related grade III oral mucositis and grade II nausea. Due to the incidental finding of a thyroid nodule at ^18^FDG‐PET scan, with normal thyroid function, an ultrasound and Doppler were performed. Ultrasound findings were consistent with the diagnosis of thyroid adenoma, which required annual follow‐up.

^18^FDG‐PET was performed after 6 cycles and after the completion of 12 cycles of therapy; at both time points, complete response (CR) was shown (Deauville score 1 and 1, respectively).

**Figure 1:**
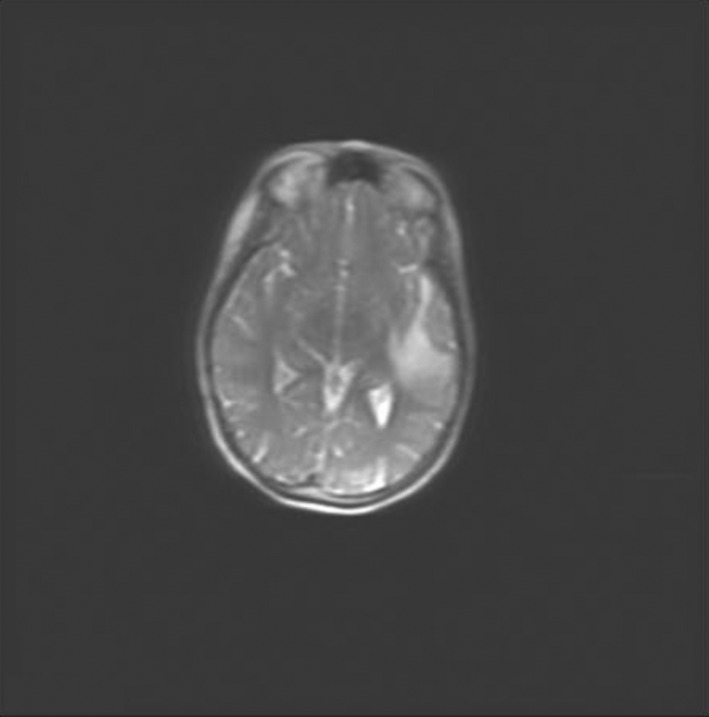
Magnetic resonance imaging at central nervous system lymphoma recurrence

In April 2017, 3 months after the completion of chemoimmunotherapy, the patient started complaining transient dysarthria and expressive aphasia; therefore, a cranial CT scan was immediately performed, showing a left temporal mass with associated edema. Magnetic resonance imaging (MRI) confirmed the finding (Figure 1), and a total body CT scan and ^18^FDG‐PET did not show further sites of disease involvement.

Thus, the patient underwent partial avulsion of the lesion through a left craniotomy at neurosurgical facility. Histological analysis revealed a diffuse large B‐cell lymphoma (DLBCL), which was considered to be consistent with a CNS recurrence of the primary mediastinal lymphoma.

The patient was treated with 4 cycles of chemoimmunotherapy with MATRix regimen.^6^

During the cycles, the patient experienced febrile episodes with negative blood cultures, responsive to broad spectrum antibacterial therapy, and an episode of blurred vision during the second cycle, with the finding of keratoconjunctivitis sicca at ophthalmic examination. After the second cycle, the patient presented a febrile episode associated with a chest CT scan finding consistent with invasive fungal infection; therefore, a broncho‐alveolar lavage was performed, showing galactomannan positivity, and an antifungal therapy with amphotericin B was undertaken.

During the 4 cycles of MATRix treatment, the patient developed grade IV neutropenia, which resolved after granulocyte‐colony stimulating factor (G‐CSF) administration, grade III anemia, which required red blood cell transfusions, and grade IV thrombocytopenia, which required platelet transfusions.

After the second cycle, an attempt to mobilize peripheral blood stem cells (PBSC) was performed, without the obtainment of a PBSC count sufficient for collection; mobilizing treatment with granulocyte‐colony stimulating factor and plerixafor was therefore performed again after the third cycle, with the achievement of a CD 34+ cells'peak of 10.000/mL on day +19 after the completion of chemotherapy and subsequent collection of 3.54 × 10^6^ CD34+ cells/kg.

The last cycle was complicated by a reaction to cytarabine, characterized by fever, skin rash, and severe headache; the subsequent cytarabine and thiotepa administrations were therefore suspended. A single, total dose of 2900 mg of cytarabine was delivered in the fourth cycle, before the development of hypersensitivity reaction.

**Figure 2:**
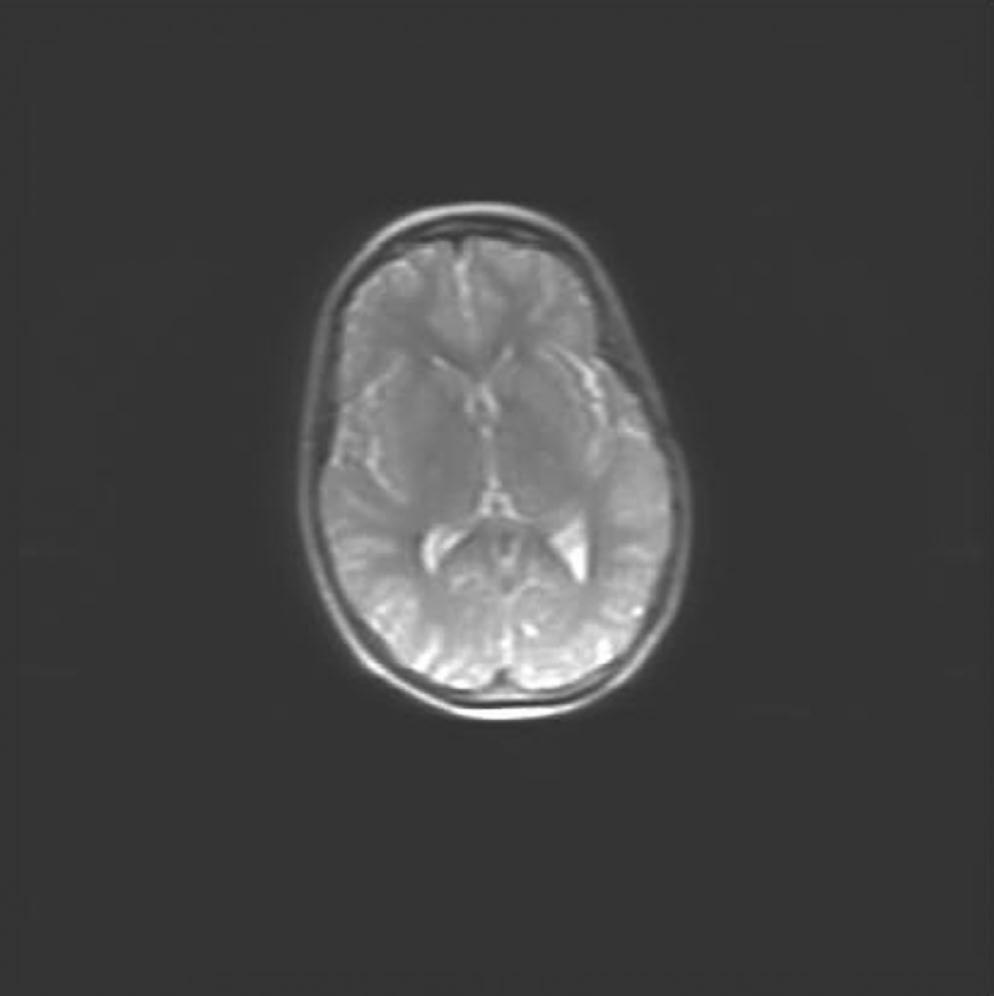
Magnetic resonance imaging at the end of MATRix (methotrexate, cytarabine, thiotepa, and rituximab) regimen (complete response)

After fourth and last cycle of therapy, both MRI (Figure 2) and ^18^FDG‐PET (Deauville score 1) showed a CR. Subsequently, the patient received conditioning treatment with carmustine and thiotepa, followed by ASCT. The main toxicities of this therapy were grade III oral and oropharyngeal mucositis associated with herpes simplex virus 1 reactivation, which required total parenteral nutrition, antiacid, and antiviral treatment, and a prolonged hematologic toxicity. Engraftment of neutrophils (ANC > 500/mmc) was observed on day +10 after ASCT, while engraftment of platelets (PLT count > 20.000/mmc) was observed on day +16. Both ^18^FDG‐PET and CT scan of the brain showed a CR 2 months after ASCT.

The case report study was approved by the local ethical committee. Written informed consent for publication of her clinical details and clinical images was obtained from the patient. Timeline of the case report is shown in Table 1.

**Table 1:** Timeline

October 2016	Diagnosis of PMBCL	
October 2016	R‐MACOP‐B	CR
April 2017	CNS recurrence	
April 2017	Start of MATRix regimen	
June 2017	Mobilization of peripheral blood stem cells	
August 2017	End of MATRix regimen	CR
January 2018	ASCT	
March 2018	Evaluation post‐ASCT	CR

Abbreviations: ASCT, autologous stem cell transplant; CNS, central nervous system; CR, complete response; MATRix, methotrexate, cytarabine, thiotepa, and rituximab; PMBCL, primary mediastinal B‐cell lymphoma; R‐MACOP‐B, rituximab, methotrexate, doxorubicin, cyclophosphamide, vincristine, prednisone, bleomycin.

PMBCL is an uncommon tumor which constitutes about 2%‐3% of all non‐Hodgkin lymphomas and 6%‐10% of DLBCL.^2^ In this disease, CNS relapse is a quite uncommon event, which constitutes nevertheless a therapeutic challenge due to its dismal prognosis. Since in literature there are currently no data available concerning management of CNS relapse in PMBCL, we refer to studies conducted in DLBCL.

In a large trial conducted in prerituximab era involving 899 patients, a cumulative CNS relapse incidence of 2.8% was reported among patients affected by intermediate or high‐grade lymphoma, who were treated with polichemotherapy regimens. In this trial, CNS recurrence appeared to occur early (all cases occurred within 24 months from the end of treatment) and was characterized by a dismal prognosis.^8^

The addition of monoclonal antibody rituximab to polichemotherapy regimens such as CHOP (cyclophosphamide, doxorubicin, vincristine, prednisone) conferred an advantage in terms of risk of CNS involvement in DLBCL, although its real benefit is still controversial.^9^

Several attempts were made to better identify patients at high risk of CNS involvement, in order to guide specific diagnostic workup and to select patients for appropriate therapeutic or prophylactic measures. Recently, an international group developed a risk model called “CNS‐International Prognostic Index” (CNS‐IPI).^10^ The score was developed analysing patients who were enrolled in studies from the German High‐Grade Non‐Hodgkin Lymphoma Study Group and the MabThera International Trial and was subsequently validated in an independent data set from the British Columbia Cancer Lymphoid Cancer database. The study confirmed the infrequence, earliness, and aggressive course of CNS relapse in aggressive B‐cell lymphomas; the model defining the CNS‐IPI included IPI factors. Of note, in the validation study, patients affected by PMBCL were excluded; another limitation of the study is the fact that, even with this robust risk score, a very high‐risk group remains difficult to identify.^10^ At diagnosis, our patient had kidney and adrenal gland involvement, more than one extranodal site, stage IV disease and elevated LDH; therefore, she had high‐risk disease (CNS‐IPI 4). Due to the lack of consensus and of recommendations on CNS prophylaxis in PMBCL and the failure of several studies to demonstrate a benefit of intrathecal (IT) prophylaxis in DLBCL, we did not perform IT prophylaxis after first‐line chemoimmunotherapy.^11^, ^12^

Since the patient achieved a complete remission after first‐line treatment, we did not perform consolidative mediastinal radiotherapy (RT); in fact, as recent studies suggested that consolidation with RT can be safely omitted in patients who achieve PET‐negativity after first‐line chemoimmunotherapy, we do not usually administer RT in this setting of patients.^13^, ^14^, ^15^

Patients who experience CNS relapse have a poor prognosis; thus, they should receive treatment according to modern protocols for primary CNS lymphoma, such as methotrexate‐/cytarabine‐based schemes. Recently, in an international randomised phase II trial, the addition of rituximab and thiotepa to a cytarabine and methotrexate therapeutic scheme showed a significant benefit in terms of CR rate, overall response rate, 30 months‐progression‐free survival and overall survival, if compared to methotrexate and cytarabine or with methotrexate, cytarabine and rituximab in patients affected by primary CNS lymphoma. In this study, patients with responsive or stable disease after the first stage were randomised to receive whole‐brain radiotherapy (WBRT) or ASCT; the results from the second randomisation showed that WBRT and ASCT are both feasible and effective as consolidation therapies.^6^, ^7^

Following the promising results showed in the study by Ferreri and colleagues, at the time of CNS relapse, we treated our patient with four courses of chemo‐immunotherapy with methotrexate, cytarabine, thiotepa, and rituximab according to MATRix protocol, with the obtainment of a CR since the second cycle of treatment; although both ASCT and WBRT are effective consolidation strategies in patients affected by primary CNS DLBCL^7^, due to the young age of the patient and the known risk of cognitive impairment related to WBRT, we decided to perform ASCT. ^18^FDG‐PET and CT scan of the brain documented the maintenance of CR two months after the procedure.

In conclusion, we report the successful management of a CNS relapse of PMBCL with MATRix treatment; further follow‐up is needed to determine long‐term outcome.

## CONFLICT OF INTEREST

None declared.

## AUTHOR CONTRIBUTIONS

MM, BC, AB, and PLZ: analyzed and interpreted the patient data regarding the hematologic disease; MC: provided advice for treatment and analyzed and interpreted the patient data regarding the hematologic disease; MM, LA, and PLZ: were major contributor in writing the manuscript; and all authors: read, revised, and approved the final manuscript.
